# Traumatic abdominal wall hernia as a component of the seatbelt syndrome: A case report of complete abdominal wall muscle transection

**DOI:** 10.1016/j.ijscr.2024.110017

**Published:** 2024-07-09

**Authors:** I. Gómez-Torres, R.L. Gamón-Giner, P.D. Menor-Duran, M. Queralt-Escrig, G. Jara-Benedetti, E. Alcobilla-Ferrara

**Affiliations:** Abdominal Wall Surgery Unit, General and Digestive Surgery Department Hospital General Universitari de Castelló, VLC, Spain

**Keywords:** Traumatic abdominal wall hernia, Abdominal wall avulsion, 3D reconstruction, Seat belt syndrome, Case report

## Abstract

**Introduction and importance:**

Seat belt syndrome (SBS) is a rare condition described as injuries sustained due to thoracic, abdominal, and pelvic compression in the context of traffic accidents. These injuries can range from minor skin abrasions to large lesions of internal organs and spinal cord involvement. Traumatic abdominal wall hernias (TAWH) are one of the injuries that can be associated.

**Case presentation:**

A 21-year-old male suffered a severe injury, resulting in complete transection of all abdominal wall musculature due to SBS, with associated visceral injury. Emergency surgery included intestinal and sigmoid colon resection, along with cava vein repair. After a prolonged recovery, a second-stage surgery for abdominal wall reconstruction was planned. Prehabilitation involved botulinum toxin and pneumoperitoneum, with surgical planning utilizing CT scan and 3D reconstruction. The second-stage surgery included transversus abdominis release and placement of double mesh.

**Clinical discussion:**

Managing traumatic abdominal wall hernias in polytrauma patients necessitates emergent surgery for vital injuries, while reconstructive surgery timing is crucial, with patient preparation being essential. Surgical planning, including 3D reconstructions, enhances accuracy, and safety, with repair technique selection depending on anatomical features. Given our patient's athletic background and preoperative vascular CT findings, flapless reconstructive surgery was chosen to mitigate vascular risks.

**Conclusion:**

The therapeutic approach to traumatic abdominal wall injuries should be individualized to each patient, with a focus on addressing vital injuries first and considering abdominal wall reconstruction surgery at a subsequent stage. Utilizing CT scan with 3D reconstruction can be a valuable tool for preoperative planning in cases involving significant abdominal wall defects.

## Highlights

• Seat belt syndrome: trauma from compression, leading to internal injuries and traumatic hernias
• Polytraumatic patient initial treatment: prioritize life-threatening injuries; address abdominal wall later
• CT scan with 3D images can aid planification for abdominal wall surgery with large muscle defects.
• Patient prehabilitation ensures optimal preoperative condition and postoperative outcomes.
• Consider patient traits, needs, and anatomy conditions when choosing surgical technique

## 1 Introduction

High-speed traffic accidents are a frequent cause of abdominal wall injuries and can also result in visceral injuries. The use of a seat belt can be involved in the injury mechanism in what is known as the seat belt syndrome (SBS).

Avulsion is a specific and rare type of injury in traffic accidents that involves the separation of tissues, such as muscle or bone. In this type of injury, present in 1 % of polytrauma patients, there is a muscle injury that is generally accompanied by injuries to the hollow viscera or splenic lacerations, which requires emergency surgery in up to one-third of cases, with an associated mortality of up to 10 % depending on the accompanying internal injuries. [[1], [2], [3]]

The indication for emergency or deferred surgery depends on several factors, including the severity of the injuries, the patient's condition, and the available resources [4,5].

Immediate surgery is crucial for life-threatening conditions like massive abdominal hernias or organ damage, preventing rapid blood loss or multi-organ failure. Delayed surgery may be suitable for less severe injuries or patients with other urgent issues, allowing stabilization and planning. However, it is recommended to be performed within a period of less than 1 year to avoid complications from muscle retraction, if the patient permits, guided by experienced surgical teams based on the patient's needs. [6,7]

Given the limited evidence, we present a severe case involving complete abdominal wall transection due to trauma. The reconstruction was performed in two stages, using 3D imaging for surgical planning optimization. Additionally, we conducted a retrospective review of cases in our institution involving patients diagnosed with traumatic injury or herniation associated with seat belt syndrome.

This work has been reported in line with the SCARE criteria [8].

## 2 Case presentation

We present the case of a 21-year-old male who was transported to the emergency room after a front-end collision with a motor vehicle. The accident occurred at 80 km/h with the seat belt properly positioned in two parts, shoulder, and abdomen.

Upon arrival at the emergency room, the patient was evaluated according to the guidelines for the management of the polytraumatized patient with a systematic approach based on the priorities indicated by the Advanced Trauma Life Support (ATLS).

**Fig. 1:**
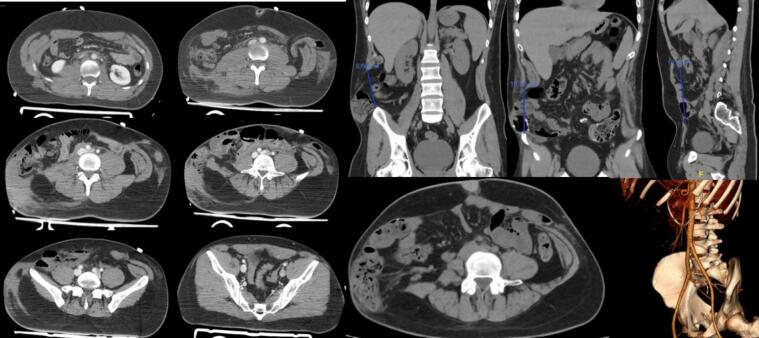
Emergency abdominal CT scan performed before urgent surgery. Vascular reconstruction.

After the primary assessment, which showed no alterations in A, B, C, or D, external injuries were examined, revealing cutaneous erosion on the thoracic and abdominal regions consistent with a seatbelt pattern. Complementary tests included a full-body CT scan, revealing complete transection of both rectus abdominal muscles and the lateral musculature of the abdominal wall, along with avulsion of the iliac spine. Additionally, intra-abdominal injuries such as hematoma of the vena cava, and injuries to the intestinal and sigmoid colon were observed (Fig. 1).

Given the findings, after 30 min of initial evaluation, urgent surgery is decided upon. A wide supra-umbilical midline laparotomy is performed for small bowel and colon resection, and vascular reparative surgery with a primary repair of the vein cava lesion, along with provisional abdominal closure, muscle approximation, and cutaneous closure without repairing the abdominal wall due to hemodynamic instability.

During the postoperative period, the patient experiences respiratory distress due to an acute process and respiratory infection, compounded by muscle weakness acquired during a prolonged ICU stay and respiratory desynchrony from complete abdominal wall muscle transection, necessitating breathing assistance.

After 6 months of fully recovering, planning for abdominal wall reconstructive surgery begins. A preoperative CT scan identifies a significant defect in the abdominal wall, including complete transection of the right lateral musculature, both rectus abdominal muscles, and partial transection of the left abdominal musculature. The absence of the right lateral musculature results in a large global defect measuring 12 cm cranio-caudally and 25 cm in maximum transverse diameter.

**Fig. 2:**
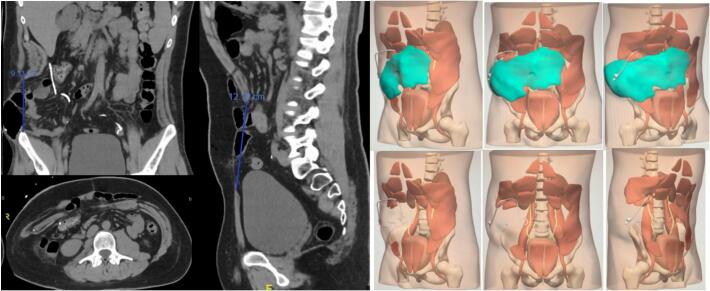
Abdominal CT scan six weeks after the administration of botulinum toxin and 3D reconstruction of the abdominal wall defect.

Due to the extensive defect and patient characteristics, myocutaneous flap reconstruction is not feasible. Instead, anatomical abdominal surgery utilizing posterior component separation and surgical prehabilitation is planned. Botulinum toxin is injected into 5 specific points in the bilateral remaining abdominal musculature (external oblique muscle, internal oblique muscle, and transverse muscle) six weeks pre-surgery to minimize the defect. Surgical planning involves 3D reconstruction of the abdominal musculature using CELLA Solutions' image creation for technique optimization (Fig. 2).

### 2.1 Surgical procedure

In the left hemiabdomen, partial injury to the internal and external oblique muscles was evident, along with a complete section of the left rectus muscle. Despite this, both anterior and posterior fascia remained intact, enabling complete anatomical repair through suturing of fascia and muscle approximation.

**Fig. 3:**
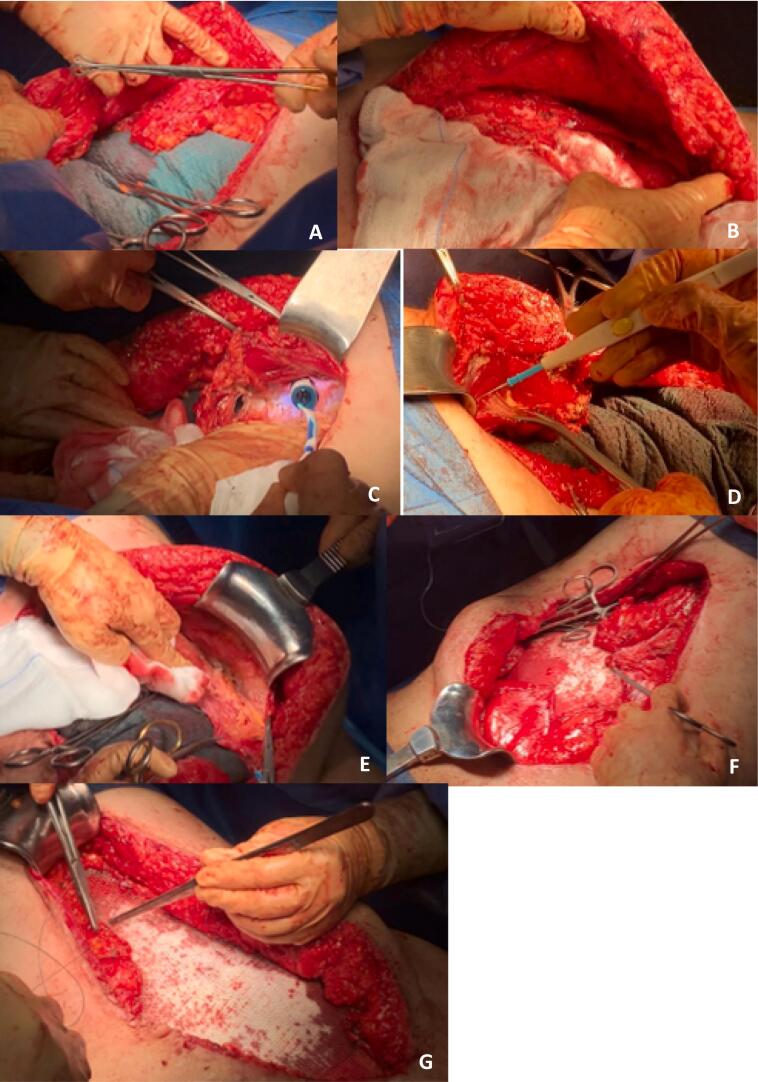
Status of abdominal musculature during surgery and steps of the surgical procedure performed. A. Right rectus abdominis muscle sectioned. B. Left rectus abdominis muscle sectioned. Lateral musculature reconstructed. C. Dissection of right Rives space. D. Dissection of left Rives space. E. Left Transversus Abdominis Release. F. Placement of biological mesh. G. Placement of polypropylene mesh.

For the right hemiabdomen, there was a complete section of both oblique muscles, the transversus abdominis muscle, and the right rectus abdominal muscle, resulting in significant muscle separation and insufficient tissue for complete anatomical repair. Therefore, bilateral posterior component separation with existing tissue was performed, necessitating the placement of a mesh bridge for reconstruction of the right lateral wall, using a biological mesh (30 × 20 cm), overlaid with a polypropylene mesh (50 × 50 cm) (Fig. 3).

### 2.2 Outcomes and follow-up

The patient spent 12 h in the ICU before being transferred to a regular ward. He experienced no complications during the hospital stay and was discharged after 7 days. At the 30-day post-surgery evaluation, no complications were noted. Functionally, muscle contraction has returned in the left hemi-abdomen, but remains limited in the right hemi-abdomen. There is no evidence of recurrence two years post-surgery. Nonetheless, the patient exhibits bulging attributed to muscular hypofunctionality, stemming from innervation injury and muscular defect. Nevertheless, the patient maintains satisfactory quality of life and has resumed an active lifestyle.

### 2.3 Retrospective review

We analyzed motor vehicle accidents involving seat belt-wearing occupants at our institution over 5 years, handling approximately 600–700 polytrauma cases annually. Among 3050 emergency department patients during this period, only 6, including the case described here, had abdominal wall avulsion, with a rate of 0.2 % or an estimated incidence of 2 per 1000. Patients averaged 40.8 years, predominantly male at a 2:1 ratio, with all cases showing muscular avulsion from the iliac spine without cutaneous injury.

Of these 6 patients, 5 needed immediate surgery for associated intra-abdominal injuries, mainly intestinal or colonic resections. Only one (patient C) had simultaneous abdominal wall repair during emergency surgery, using an anatomically guided technique.

Conversely, the other 4 patients did not have immediate abdominal wall surgery. Patient A had delayed reparative surgery, while Patients D and F underwent subsequent surgeries for intestinal transit reconstruction and await scheduled abdominal wall repair. Patient E declined surgery due to being asymptomatic.

Patient B's abdominal wall injury was diagnosed by ultrasound four weeks after the accident. He had scheduled surgery with anatomical repair and mesh placement using the Carbonell-Tatay technique.

**Fig. 4:**
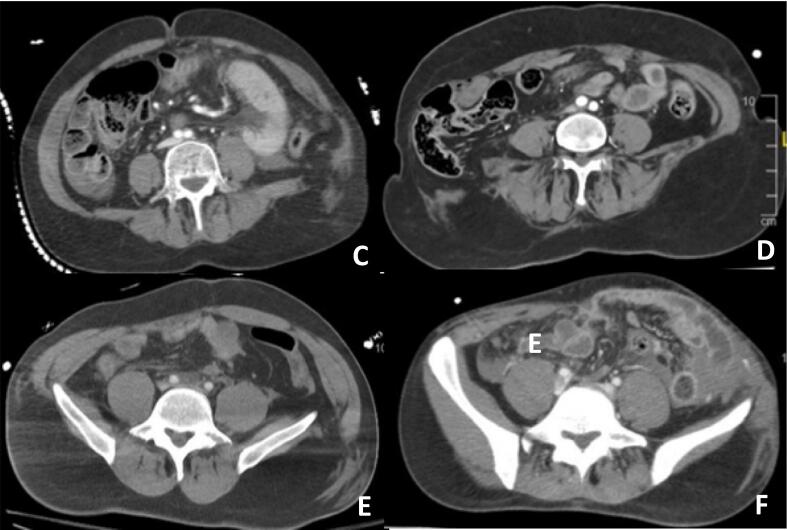
CT scan of patients C, D, E, and F revealing muscular laceration with traumatic hernia.

The characteristics of the patients are summarized in Table I. The traumatic injuries of the patients visualized by CT scan can be observed in Fig. 4.

**Table I:** Summary of patients with seat-belt-associated traumatic abdominal wall hernia.

	Age (years)	Sex (M/F)	Location of TAWH	Associated injuries	Emergency surgery	Immediate abdominal wall repair	AW delayed surgery
A^a^	21	M	Complete transection of both rectus abdominis muscles and bilateral lateral musculature	Sigmoid colon, small bowel, and cava vein	Yes^a^	No	Yes^a^
B	52	M	Right avulsion of the lateral musculature from iliac spine	No	No	No	Open surgery with mesh
C	27	M	Left avulsion of the lateral musculature from the iliac spine	Aortic dissection, retroperitoneal hematoma, hepatic laceration, sigmoid colon, small bowel, hemothorax, multiple rib fractures	Yes: small bowel resection, Hartmann procedure, hepatic packing	Yes: anatomical repair with muscular suture	No
D	28	M	Left avulsion of the lateral musculature from iliac spine	Sigmoid colon, splenic laceration, multiple limb fractures, multiple rib fractures	Yes: Hartmann procedure	No	No. Awaiting
E	59	F	Right avulsion of the lateral musculature from the iliac spine	Small bowel, psoas hematoma, aortic dissection, multiple rib fractures, kidney laceration	Yes: ileocecal resection	No	No
F	58	F	Left avulsion of the lateral musculature from iliac spine	Rectus abdominis and psoas hematoma, small bowel, retroperitoneal hematoma	Yes: small bowel resection, Hartmann procedure	No	No. Awaiting

a Patient described in the paper.

## 3 Discussion

The “seat belt syndrome” results from trauma due to seatbelt force after car accidents, affecting both drivers and passengers. Injuries associated with seatbelt use are well-documented, often affecting belt-covered areas. While mostly mild like bruises, abrasions, hematomas, or rib fractures, severe injuries can occur in a small percentage of high-speed accidents, involving intra-abdominal organs and the abdominal wall, ranging from minor hernias to severe avulsions. [9].

Firstly, the article discusses managing polytrauma patients with traumatic abdominal wall hernias, emphasizing prioritizing vital injuries during initial surgery. Abdominal wall repair is reserved for stable cases, with complex surgery possibly deferred to a later stage, as shown in the case study.

Secondly, the timing of reconstructive surgery in the second stage is crucial. Many authors advocate for early repair, depending on factors such as the type of initial surgery, contamination level, associated injuries, patient stability, and organic function [10,11]. In this case, due to the patient's complex postoperative course, surgery was postponed until full recovery and prehabilitation with botulinum toxin and pneumoperitoneum could be conducted.

Thirdly, patient preparation and prehabilitation for complex abdominal wall repair surgery are crucial. Conducting reconstructive surgery as a second step allows physical recovery before complex procedures and implementing prehabilitation techniques. Botulinum toxin enhances tissue mobility and elasticity, aiding anatomical approximation. Progressive pneumoperitoneum reduces omental adhesions to the peritoneum, facilitating surgical space dissection without visceral injury. [12]

Limited evidence supports using 3D reconstructions for surgical planning in complex abdominal wall issues. This method improves accuracy, efficiency, safety, and short-term outcomes, cutting procedural time and additional interventions, potentially lowering overall patient costs. Despite initial expenses, the accuracy and efficacy improvements justify 3D planning, possibly reducing long-term costs [13].

Lastly, the repair technique choice, with or without mesh, depends on the hernia's anatomical features. In cases of hollow viscus injuries, the decision considers peritoneal contamination risk. Biological meshes are preferred in contaminated environments [14]. Various flaps and grafts are available for large tissue defects, chosen based on defect extent, location, and patient characteristics. Myocutaneous flaps like the lateral or bilateral rectus abdominis flap, or the external oblique muscle flap, are common for inguinal defects. Flaps of the lumbar square muscle, sartorius, rectus femoris, and semitendinosus muscle can be used for defects involving the entire abdominal musculature.

Myocutaneous flaps need adequate blood supply to avoid necrosis, often relying on intrinsic circulation without lower vessel connections. In extensive defects, arterial or venous anastomoses may be needed. In specific cases, myocutaneous flaps with revascularization techniques, like the rectus abdominis flap with an epigastric pedicle or latissimus dorsi muscle flap, may be used. [15].

Considering our patient's athletic background and preoperative vascular CT showing complete epigastric vessel transection, we opted for reconstructive surgery without flaps. This decision was influenced by the risk of inadequate vascularization to a limb flap.

The possible long-term complications of patients with traumatic abdominal wall avulsion should be taken into account. Despite reparative surgery, they may experience extensive functional limitations due to denervation of the abdominal musculature, as well as chronic pain from reconstructive surgery and mesh, in addition to the high risk of incisional hernia recurrence.

## 4 Conclusions

Traumatic abdominal wall hernias (TAWH) secondary to seatbelt use in polytraumatic patients are rare but should be considered after car accidents. Treatment requires a multidisciplinary surgical approach, addressing vital injuries first and considering abdominal wall reconstruction later. Surgical preparation and planning should be tailored to each patient's anatomy and abdominal wall defect. 3D reconstruction technology can aid in surgical planning, particularly for complex cases, although its high cost limits its use. The choice of surgical technique should be personalized based on the patient's characteristics, priorities, and the extent of the defect.

## Informed consent statement

Written informed consent was obtained from the patient for publication and any accompanying images. A copy of the written consent is available for review by the Editor-in-Chief of this journal on request.

## Additional information

This study has not been previously published elsewhere for publication and it will not be sent to another journal until a decision will be made concerning publication.

## Research quality

The authors followed the SCARE guideline, during the conduct of this report.

## Ethical approval

According to the guidelines of the Ethics Committee of HGUCS (Research and Innovation Commission of Castelló (CI2C)), ethics approval is not required for case reports or case series deemed not to constitute research at our institution.

## Funding

This research received no external funding.

## Author contribution

IGT wrote and edited the paper. All authors: RLGG, PDMD, MQE, GJB and EAF reviewed the paper and revised it critically for intellectual content. Each author has participated sufficiently in the work of reviewing and approving the study. All authors also contributed to the surgical and clinical management of the patient.

## Guarantor

All authors of the article have approved its publication and supervised its writing.

## Research registration number

Not ‘first in man’ study.

## Conflict of interest statement

All authors have no conflicts of interest or financial disclosures.

## Data Availability

The datasets generated during and/or analyzed during the current study are available from the corresponding author on reasonable request.
